# Supplementary material for: Hippocampal Atrophy in Pediatric Transplant Recipients with Human Herpesvirus 6B
Source: Microorganisms. 2021 Apr 8;9(4):776. doi: 10.3390/microorganisms9040776 (PMC8068176; doi:10.3390/microorganisms9040776)
Supplement: Supplementary file 1 [file microorganisms-09-00776-s001.pdf]

Table S1. Magnetic resonance imaging acquisition

Patient	HHV-6B infection	Before transplantation				After transplantation			
		MRI scanner	TR (ms)	TE (ms)	Thickness (mm)	MRI scanner	TR (ms)	TE (ms)	Thickness (mm)
**1**	+	Canon, Atlas SPEEDER, MRT-200, 1.5T	4500	105	6	Canon, Atlas SPEEDER MRT-200, 1.5T	4500	105	6
**2**	+	Siemens, MAGNETOM Trio, 3T	5210	72	5	Siemens, MAGNETOM Trio 3T	5210	72	5
**3**	+	Canon, Atlas SPEEDER, MRT-200, 1.5T	4500	105	6	Siemens, MAGNETOM Avanto 1.5T	4500	105	6
**4**	+	Siemens, MAGNETOM Trio, 3T	5210	72	5	Siemens, MAGNETOM Trio 3T	5210	72	5
**5**	+	Siemens, MAGNETOM Aera, 1.5T	3800	90	6	Canon, Atlas SPEEDER MRT-200, 1.5T	3800	90	6
**6**	+	Siemens, MAGNETOM Verio, 3T	5210	69	5	Siemens, MAGNETOM Trio 3T	5210	69	5
**7**	+	Siemens, MAGNETOM Verio, 3T	5210	69	5	Siemens, MAGNETOM Verio 3T	5210	69	5
**8**	+	Siemens, MAGNETOM Avanto, 1.5T	3800	91	6	Siemens, MAGNETOM Avanto 1.5T	3800	91	6
**9**	-	Siemens, MAGNETOM Trio, 3T	6500	69	5	Siemens, MAGNETOM Trio 3T	6500	69	5
**10**	-	Siemens, MAGNETOM Trio, 3T	5200	131	5	Siemens, MAGNETOM Trio 3T	5200	131	5
**11**	-	Siemens, MAGNETOM Trio, 3T	5210	69	5	Siemens, MAGNETOM Trio 3T	5210	69	5

**12**	-	Siemens, MAGNETOM Trio, 3T	5210	69	5	Siemens, MAGNETOM Trio 3T	5210	69	5
**13**	-	Siemens, MAGNETOM Trio, 3T	6590	131	5	Siemens, MAGNETOM Verio 3T	6590	131	5
**14**	-	Siemens, MAGNETOM Trio, 3T	5210	72	5	Siemens, MAGNETOM Trio 3T	5210	72	5
**15**	-	Siemens, MAGNETOM Trio, 3T	6600	131	5	Siemens, MAGNETOM Trio 3T	6600	131	5
**16**	-	Siemens, MAGNETOM Trio, 3T	5210	72	5	Siemens, MAGNETOM Trio 3T	5210	72	5
**17**	-	Siemens, MAGNETOM Trio, 3T	5210	72	5	Siemens, MAGNETOM Trio 3T	5210	72	5
**18**	-	Siemens, MAGNETOM Trio, 3T	5620	131	5	Siemens, MAGNETOM Trio 3T	5620	131	5
**19**	-	Siemens, MAGNETOM Trio, 3T	5200	131	5	Siemens, MAGNETOM Trio 3T	5200	131	5
**20**	-	Siemens, MAGNETOM Trio, 3T	5210	72	5	Siemens, MAGNETOM Trio 3T	5210	72	5

Abbreviation: HHV-6 = human herpesvirus 6; TR = repetition time; TE = echo time.
